# Expansion and functional diversification of a leucyl aminopeptidase family that encodes the major protein constituents of *Drosophila *sperm

**DOI:** 10.1186/1471-2164-12-177

**Published:** 2011-04-05

**Authors:** Steve Dorus, Elaine C Wilkin, Timothy L Karr

**Affiliations:** 1 Department of Biology and Biochemistry, University of Bath, Bath BA2 7AY, UK; 2 Centers for Evolutionary Medicine and Informatics and Infectious Diseases and Vaccinology, Biodesign Institute, Arizona State University, Tempe, AZ 85287-5001, USA

**Keywords:** sperm, proteomics, gene duplication, gene family, protease, spermatogenesis, testis

## Abstract

**Background:**

The evolutionary diversification of gene families through gene creation (and loss) is a dynamic process believed to be critical to the evolution of functional novelty. Previous identification of a closely related family of eight annotated metalloprotease genes of the M17 Merops family in the *Drosophila *sperm proteome (termed, Sperm-LeucylAminoPeptidases, S-LAPs 1-8) led us to hypothesize that this gene family may have experienced such a diversification during insect evolution.

**Results:**

To assess putative functional activities of S-LAPs, we (i) demonstrated that all S-LAPs are specifically expressed in the testis, (ii) confirmed their presence in sperm by two-dimensional gel electrophoresis and mass spectrometry, (iii) determined that they represent a major portion of the total protein in sperm and (iv) identified aminopeptidase enzymatic activity in sperm extracts using LAP-specific substrates. Functionally significant divergence at the canonical M17 active site indicates that the largest phylogenetic group of S-LAPs lost catalytic activity and likely acquired novel, as yet undetermined, functions in sperm prior to the expansion of the gene family.

**Conclusions:**

Comparative genomic and phylogenetic analyses revealed the dramatic expansion of the S-LAP gene family during *Drosophila *evolution and copy number heterogeneity in the genomes of related insects. This finding, in conjunction with the loss of catalytic activity and potential neofunctionalization amongst some family members, extends empirical support for pervasive "revolving door" turnover in the evolution of reproductive gene family composition and function.

## Background

Gene duplication is considered a fundamental process in the generation of genes with novel functions and ultimately the evolution of biological diversity [1]. A wide variety of evolutionary and comparative genomic analyses have been used to infer the origins and fates of newly created genes [2-5]. While more limited in scope, molecular genetic and functional studies [6-9] have provided new insights into the role gene duplication has played in generating novel functions. In Drosophila, comparative genomic analyses have revealed an enrichment of rapidly evolving or lineage specific gene families associated with reproduction [10]. While the functional significance of this remains to be fully determined, the trend can be explained, in part, by creation of male-biased genes through retrotransposition. Recent studies have demonstrated that retrogenes in both Drosophila [11,12] and primates [13-16] tend to acquire testis-specific expression. However, we still have limited understanding of why new genes frequently acquire testis expression and whether testis-specific expression is obtained prior to, or following, acquisition of functionality. Nonetheless, several studies suggest that newly generated genes and gene families have had a functional impact on spermatogenesis and on the evolution of sperm [8,12,17].

Although new testis-specific genes might be expected to have gained functions in spermatogenesis, there are a limited number of characterized examples of such genes in *Drosophila*. One example is the strict paternal-effect gene *ms(3)K81 *(termed "*K81*"), a gene created by retrotransposition prior to the divergence of the melanogaster subgroup [8]. In wild-type eggs fertilized by sperm from K81 males, paternal chromosomes systematically fail to properly separate sister chromatids during the first zygotic division leading to lethality early in embryogenesis [8]. Interestingly, *K81 *is expressed only in primary spermatocytes where it presumably regulates aspects of spermatogenesis required for proper sperm function in the egg. A second example is *mojoless *(*mjl*), created approximately 50mya through the retrotransposition of *shaggy *(*sgg*), a glycogen synthase kinase-3 encoding gene [9]. RNAi knockdown of *mjl *resulted in loss of the male germ line and male sterility. Although not yet ascribed a characterized function in the testis or sperm, a third gene, *Sdic*, is a newly created gene encoding a protein present in the sperm tail [17]. *Sdic *is an unusual case of an X-linked chimeric gene specific to Drosophila melanogaster that was created through the duplication of *annexinX *(*AnnX*) and subsequent fusion with *Cdic*, a cytoplasmic dynein gene.

The application of mass spectrometry (MS) to the study of sperm has provided our first insights into the dynamic role new gene creation has played in shaping the constituents of the sperm proteome [18] and has raised intriguing questions about how these new genes have impacted the molecular evolution of sperm. For example, analysis of the Drosophila sperm proteome revealed 3 novel sperm genes specific to *D. melanogaster *and a further 4 sperm genes created through retrotransposition during Drosophila evolution [12]. Amongst these is the retrogene *CG13340*, which encodes S-LAP 7, examined in this study. It is noteworthy that these new sperm components were found to be proteins across diverse functional classes, and both rapidly evolving (in the case of protamine genes, *Mst35a *and *Mst35b*) and highly conserved (X-linked *Tektin *gene cluster).

Many proteolysis-related genes (including peptidases, proteases and inhibitors of proteolysis) have reproductive functions across diverse taxa, including insects [19-21] and mammals [22]. Amongst these, proteolysis-related genes expressed in the Drosophila accessory gland have been particularly well studied in terms of their mediation of a wide range of effects on females (see [23] for a recent review). Proteins with proteolytic activity are also found in the *Drosophila *female reproductive tract and a subset of these are encoded by recently created genes [24]. In both mammals and *Drosophila*, the genes involved in reproductive proteolytic pathways or their targets have been demonstrated to evolve rapidly [22,25,26] and it is hypothesized that this is due to coevolutionary forces associated with sexual conflict (either between males and females or males and males) [27,28]. These observations have been extended to the sperm proteome where an intensified signature of positive selection was observed for membrane and acrosomal sperm proteins that included a diverse set of metalloproteases and protease inhibitors [29].

Although well documented in reproductive tissues generally, only recently have proteolytic and related genes been shown to be present in spermatozoa [18,29-31]. The functional significance and role in fertilization of these classes of proteins is unknown, but demonstration of their presence and activity in sperm is a necessary step towards more targeted studies. Here we present a detailed evolutionary and functional characterization of a family of eight Drosophila sperm leucyl aminopeptidases that we have termed Sperm-LAPs (S-LAP 1-8). Computational annotation places the S-LAPs in the M17 family of leucyl aminopeptidases [32]. Of the 10 annotated leucyl aminopeptidases in the Drosophila genome, the S-LAPs are specifically expressed in the testis and all encode proteins incorporated in mature sperm [18,33]. Here we describe results of detailed comparative genomic analyses that demonstrate an expansion and diversification of the S-LAP gene family during Drosophila evolution. We also provide (i) an independent proteomic analysis confirming the presence of S-LAPs in sperm, (ii) a biochemical analysis of S-LAPs quantity and abundance in sperm, (iii) data indicating that S-LAPs are expressed specifically in the testis and (iv) evidence confirming S-LAP enzymatic activity supporting their functional annotation. This striking specificity of expression and cellular location suggests an unknown functional requirement for aminopeptidases in sperm. Furthermore, evolutionary diversification at the active site indicates that some S-LAPs have lost enzymatic activity and that the putative neofunctionalization of these non-enzymatic S-LAPs may be related to the subsequent expansion of the gene family. These findings thus support a scenario where functional diversification within a gene family may promote further evolutionary changes in gene family composition through the selective retention of newly created genes.

## Results

### Comparative Genomics of the S-LAP family in Drosophila

**Figure 1:**
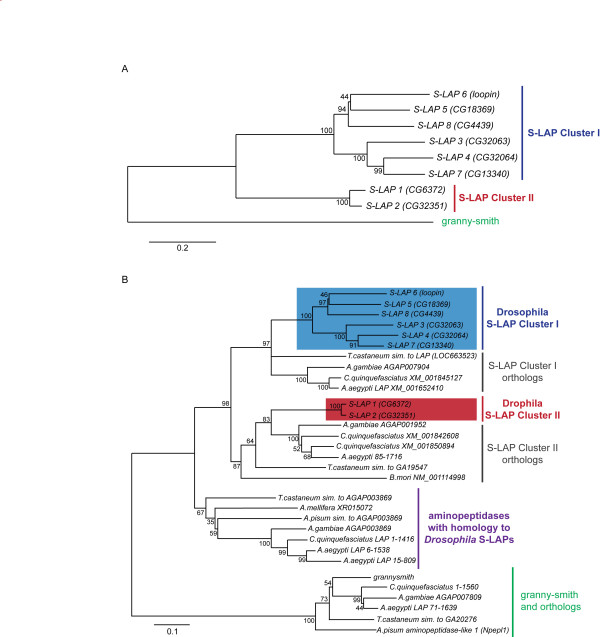
**Evolutionary relationship of S-LAPs**. (A) Phylogeny of *D. melanogaster *S-LAPs and *granny-smith*. Minimum Evolution distance methods were used to construct the bootstrap consensus protein phylogeny (1000 replicates). Bootstrap values are presented next to relevant nodes and the phylogeny is drawn to scale based on amino acid substitutions per site. This analysis revealed two related clusters of *D. melanogaster *S-LAPS, Cluster I and II. (B) Expanded S-LAP family in *Drosophila*. Comparative genomic analyses identified a total of 17 related aminopeptidases in *A. gambiae*, *C. quinquefasciatus*, *A. aegypti*, *T. castaneum*, *A. pisum *and *A. mellifera*. Minimum evolution distance methods were used to construct the bootstrap consensus protein phylogeny (1000 replicates). Bootstrap values are presented next to relevant nodes and the phylogeny is drawn to scale based on amino acid substitutions per site. S-LAP Cluster I, comprised of 6 genes in *Drosophila*, is related to a single copy aminopeptidase in mosquitos and *T. castaneum*. Similarly S-LAP Cluster II, comprised of two genes in *Drosophila*, is related to a single copy aminopeptidase in other insect taxa with the exception of *C. quinquefasciatus *where two gene copies are present in the genome. In contrast, *granny-smith *has "one-to-one" orthology relationships in other insect taxa.

Gene Ontology (GO) analysis [34] previously identified eight related leucylaminopeptidases of the M17 Merops family with an overall average amino acid identity of 53.7% [18]. We examined their evolutionary relationships by first determining that the annotated aminopeptidase, *granny-smith*, was the closest related paralog to the S-LAP gene family (17.4% average amino acid conservation with the S-LAP family). *granny-smith *has not been identified in Drosophila sperm [33] and has no reported function in spermatogenesis or reproduction. Phylogenetic analysis of *D. melanogaster *S-LAPs and *granny-smith *revealed two distinct S-LAP clusters (Figure 1a): Cluster I includes S-LAPs 3-8 genes (*CG32063*, *CG32064*, *CG18369*, *loopin*, *CG13340*, and *CG4439*, respectively) and Cluster II includes S-LAPs 1-2 genes (*CG6372 *and *CG32351*, respectively). The average synonymous divergence levels amongst the S-LAP genes (dS = 1.84) indicated that the expansion of the gene family was not recent in Drosophila evolution. All pairwise values exceeded 1.1 with the exception of CG6372/CG32351 (dS = 0.842), a finding consistent with a more recent gene duplication event resulting in Cluster II and our phylogenetic analyses (see below). To further estimate the evolutionary timing of S-LAP gene family expansion, orthologs were identified using reciprocal "best-hit" blast searches, for each S-LAP in the 12 available Drosophila genomes. This analysis determined that all S-LAPs have unique orthologs in most, if not all the Drosophila genomes and that all have distinct orthologs in *D. grimshawi*, the most ancestrally diverged Drosophila species for which genomic data is available (a phylogeny containing all identified Drosophila orthologs is provided in Additional File 1, Figure S1). Therefore, S-LAP gene family expansion either predates or occurred early in the evolution of the genus *Drosophila*, an observation consistent with the observed levels of synonymous divergence.

### Related aminopeptidase genes in other insect taxa

A comprehensive search for related aminopeptidases was conducted in other insect species using reciprocal blast searches against a dataset combining all annotated REFSEQ, non-REFSEQ and *ab initio *gene sequences in *Anopheles gambiae*, *Culex quinquefasciatus*, *Aedes aegypti*, *Tribolium castaneum*, *Nasonia vitripennis*, *Acyrthosiphon pisum*, *Bombyx mori *and *Apis mellifera*. This resulted in the identification of 17 aminopeptidases related to the S-LAP family and 6 orthologs of *granny-smith*. The number of identified S-LAP related aminopeptidase genes varied between taxa, ranging from none in *N. vitripennis *to 4 in *A. aegypti *and *A. gambiae*. Copy number determination of related aminopeptidase genes in each species was also confirmed through the analysis of genome assembly sequences in each species (data not shown).

### S-LAPs: an expanded gene family in *Drosophila*

Phylogenetic analysis of the S-LAP gene family, including related aminopeptidases from other taxa, revealed that S-LAP Cluster I and II are related to single-copy genes in most insect taxa (Figure 1b). The 6 members of Cluster I form a monophyletic group with a single related gene in all 3 mosquito species and the beetle, *T. castaneum*. This is consistent with gene family expansion during early *Drosophila *evolution. Furthermore, no Cluster I related S-LAP genes were detected in *N. vitripennis*, *A. pisum*, *B. mori *and *A. mellifera*. The two more closely related *Drosophila *members of Cluster II also form a monophyletic group with single-copy genes in other insect taxa with the exception of *C. quinquefasciatus *where two related aminopeptidases were found. These observations are in contrast to *granny-smith *that has "one-to-one" orthology relationships in all taxa where an ortholog was identified. It is also noteworthy that our analysis identified a set of seven aminopeptidases in non-*Drosophila *taxa (Figure 1b) distinct from either *Drosophila *S-LAP Cluster I or II. This includes a single copy aminopeptidase gene across 5 insect taxa and 2 putative, additional paralogs in *A. aegypti*.

### Mechanisms of new S-LAP gene creation

The age of S-LAP clusters and the associated sequence divergence at these loci means that hallmarks associated with gene duplication mechanisms are often obscured, making gene duplication mechanisms difficult to infer. However, two pairs of S-LAPS (S-LAP 1 and 2; S-LAP 3 and 4) are immediately adjacent in the genome and share common intron/exon structure suggesting they were created through tandem duplication. S-LAP 7 has previously been characterized as a retrotransposed gene [11,12]. Consistent with a retroposition event, S-LAP 7 is intronless, unlike its likely progenitor S-LAP 4, and these genes reside in different genomic regions (2R: 50C5 and 3L: 67E4, respectively).

### S-LAPs are expressed specifically in the testis

**Figure 2:**
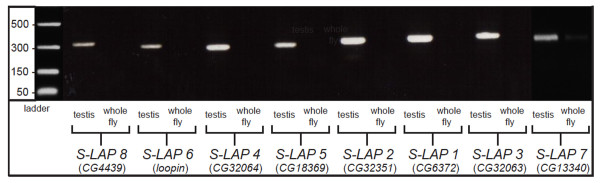
**S-LAPs are specifically expressed in testis**. RT-PCR analysis reveals testis specific expression of the *D. melanogaster *S-LAPs. Testis expression is compared to expression in the remainder of the fly using gonadectomized males.

Expression of each S-LAP in *D. melanogaster *was characterized by RT-PCR using gene specific primers. A high level of testis-specific expression for all eight S-LAPs was observed with no detectable expression in gonadectimized males (Figure 2), a finding consistent with previous microarray studies [35]. The testis-specific expression of the S-LAP family is in contrast to *granny-smith*, the closest related leucyl aminopeptidase in the genome, which is expressed broadly across a range of tissues, including (but not limited to) testis, ovary, tubule and salivary gland [35].

### Confirmation and quantification of S-LAPs in *Drosophila *Sperm by 2D-Gel

**Figure 3:**
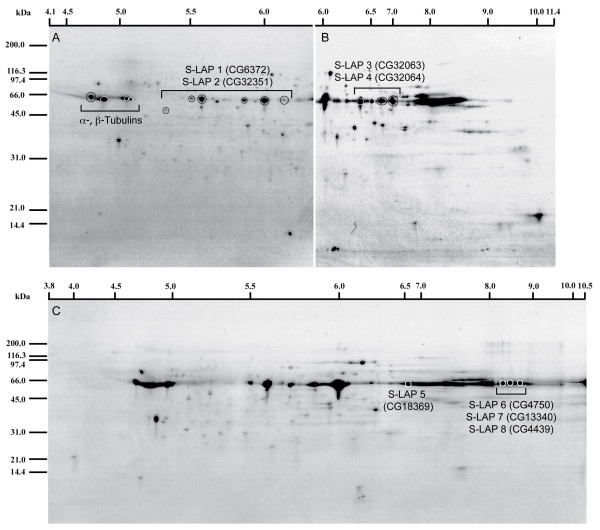
**Annotation of S-LAPs on 2D gels of purified sperm**. Gels were run using duplicate samples of solubilized sperm protein (100 ug/gel). Proteins were first separated on isoelectric focusing gels with pH ranges of (A) 4-7, (B) 6-11 and (C) 3-10 followed by second dimension SDS-PAGE using 12% Bis-Tris reducing gels. Spots were visualized using Coomassie Blue R250 and spots excised and submitted for MALDI-TOF and tandem MS/MS analyses. The α- and β-tubulins are indicated in (A) at the expected pI and molecular weight positions. Regions of the gel identified as S-LAPs are indicated by brackets with individual spots circled.

As part of an exhaustive analysis of sperm proteins from 2D-gels of varying isoelectric gradients, all eight S-LAPs were identified by both MALDI-TOF and tandem MS/MS analyses (Figure 3, Table 1). High confidence of S-LAP protein identification was supported by, (i) multiple peptide assignments for each protein (with an average of 5.8 peptides per S-LAP protein) as assigned by Scaffold (Proteome Sciences); (ii) Mowse scores from Maldi-tof measurements that exceeded statistical scoring requirements [36] and (iii) replicate spot assignments from multiple 2D-gels under varying separation conditions (Figure 3). Tubulins were also identified on 2D-gels at the expected molecular weight and isoelectric point positions (Figure 3).

**Table 1:** S-LAPs identified in *D. melanogaster *sperm by MS

Gene Name	FlyBase ID	Chromosome (Cytological Location)	Peptide Fragments Identified by Whole Sperm Tandem MS (# of times identified)*	Confirmed Mascot ID**
S-LAP 1 (CG6372)	FBgn0035915	3L (66D8-66D8)	Y.YSVVAVVGLGK.E R.FPLWNFYSK.A (2) R.LLFAVEPER.I R.IFVPAIDLYATK.D	Yes

S-LAP 2 (CG32351)	FBgn0052351	3L (66D8-66D8)	R.GDEFEYLR.E (2) R.ISVPSIDLYATK.D (4) R.VPYYSAVAVIGLGK.E R.FPLWNYYSK.A	Yes

S-LAP 3 (CG32063)	FBgn0045770	3L (67E3-67E4)	K.VFNNVDSEFR.S K.GIVLGLYEK.E R.IAAGIGAR.S K.GIVLGLYEK.E K.LELHGSPDVESWTR.G (4)	Yes

S-LAP 4 (CG32064)	FBgn0052064	3L (67E4-67E4)	R.LVVDVATLGSGVK.K K.QITDEMGYDLSNDGR.G (2) K.QFQSAGALTGDR.V K.IFNNIDPEFR.S K.PGDVVTLMNHK.S K.GLVVGVYQK.E K.LELYESPDYEGWTR.G (2) K.GITFNSGAMNLR.K	Yes

S-LAP 5 (CG18369)	FBgn0033860	2R (50B4-50B5)	R.HNPLPYLLKDR.M R.VAAGVGAR.A R.ETGIKGELGK.G K.QLVTPNLTFDISNR.G	Yes

S-LAP 6 (loopin)	FBgn0259795	2R (53C9-53C10)	K.QGAAYNENEELDEGMENVR.V (5) N.AVTLDDALGGK.L R.GTGMTTTINPR.P K.TTANAVTLDDALGGK.L (3) K.GVVVGVYTK.D	Yes

S-LAP 7 (CG13340)	FBgn0033868	2R (50C5-50C5)	R.LVVDIATLNTGVK.K (2) R.LPLWQYYR.R K.YGLVPYLTK.K K.GVVVGLYQK.E	Yes

S-LAP 8 (CG4439)	FBgn0034132	2R (53C6-53C6)	K.LELYGSTDQDAWTR.G (5) K.HGVPPYLLK.D (3) R.LYQNIDK.E K.ESGLNGELGVGR.L K.EGAGFNAEEVIDEGMENVR.V (2)	Yes

*MS analysis from [18].

** 2D-gel protein spot locations presented in Figure 3.

Visual inspection of 2D-gels clearly indicated that both the S-LAPs and tubulins represent a substantial proportion of the total protein in sperm on 2D-gels (brackets, Figure 3). We determined the relative abundance of each by comparing spot signal intensity for each class. Five 2D-gels (pI range 4-7, Figure 2) were processed, scanned and protein spot intensities calculated. The results indicate an approximate 2-fold abundance of S-LAPs (Table 2) over tubulins. Thus, we conclude that the tubulins and the S-LAP family represent the major mass of protein in sperm.

**Table 2:** Relative levels of tubulins and S-LAPs from 2D gels.

	**Spot Intensity (arbitrary units × 10**^**6**^**)**					
**2D-gel**	**Tubulin**	**S-LAPs**	**Gel Total**	**Tubulin/total (%)**	**S-LAP/total (%)**	**LAP/Tubulin**

Gel 1	2.42	5.00	50.40	4.80	9.93	2.07

Gel 2	1.42	3.23	39.50	3.58	8.19	2.28

Gel 3	2.03	3.68	43.26	4.69	8.51	1.81

Gel 4	2.01	3.88	52.10	3.87	7.44	1.92

Gel 5	1.90	5.36	66.02	2.89	8.12	2.81

			**Average**	3.97	8.44	2.18
			
			**Median**	3.87	8.19	2.07
			
			**S. D. (+/-)**	0.80	0.92	0.34

### S-LAP enzymatic activity in purified sperm

S-LAP activity in sperm extracts was directly measured using L-Leu-AMC, a sensitive and specific fluorogenic substrate for leucine aminopeptidase activity [37]. Negligible activity was observed in intact sperm suggesting that S-LAPs were not exposed on the sperm surface (L-Leu-AMC is not permeable to intact cells). Sonication of sperm in buffer alone resulted in measurable S-LAP activity but with low protein yield (Table 3). Addition of DTT resulted in both higher levels of soluble protein and measurable S-LAP activity but with reduced specific activity (Table 3). However, while this establishes presence of leucylaminopeptidase activity, the relative contribution of each S-LAP to the observed signal remains unclear.

**Table 3:** Leucine aminopeptidase activity in purified *Drosophila *sperm

Sperm Treatment	Protein (ug)	% total*	Activity (mU)	S.A.‡ (U/mg)
Buffer	<0.25	0.6	0	0

Buffer + sonication	1.1	2.2	1.78	1.62

Buffer+DTT+sonication	5.4	10.8	4.39	0.81

*The percent values are calculated from maximal amount of extractable sperm protein.

‡ Specific activity.

### Divergence at cation ligand and catalytic residues

**Figure 4:**
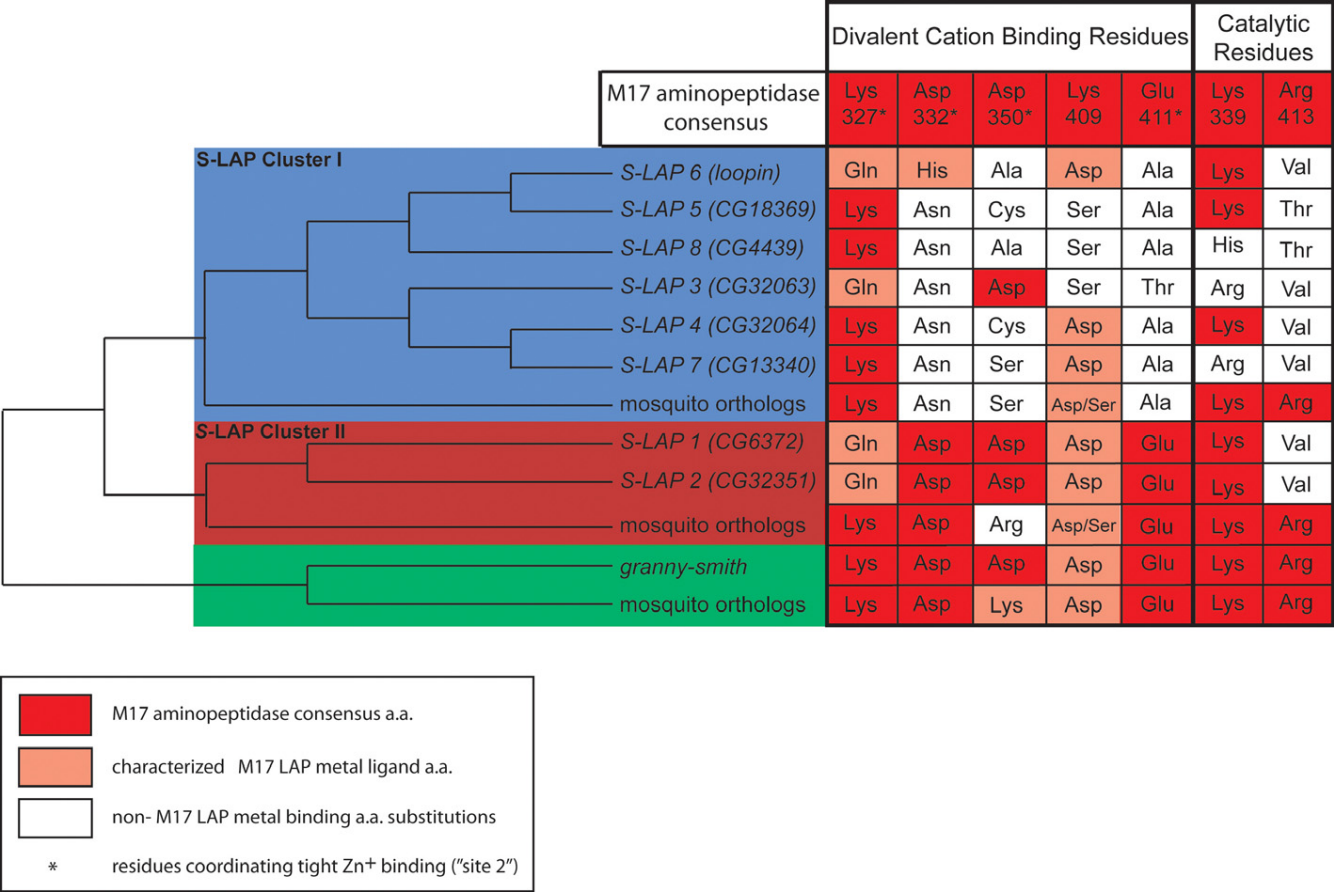
**S-LAP divergence at divalent cation binding and catalytic residues**. Amino acid composition at the seven residues involved in metal ion binding and catalytic residues is displayed according to the S-LAP phylogeny presented in Figure 1. For cation binding sites, residues matching the M17 leucyl aminopeptidase consensus are highlighted in red, substitutions to other metal binding amino acids in M17 leucyl aminopeptidases are highlighted in pink and substitutions to non-M17 leucyl aminopeptidase metal binding residues in white. For catalytic residues, residues matching the M17 leucyl aminopeptidase consensus are highlighted in red and those divergent from the consensus in white. Amino acid residues Lys327, Asp332, Asp350 and Glu411 comprise the tight, Zn2+-specific binding (site 2), while residue 409 is involved in loose coordination of divalent cations (site 1).

Biochemical and x-ray crystallography studies have defined 7 primary active site residues in M17 leucyl aminopeptidases involved in catalysis and binding of divalent cations, including a critical Zn^2+ ^ion binding region [38,39]. These sites are highly conserved in leucyl aminopeptidases across animal taxa strongly suggesting their critical role in the coordination of the metal ions, water and a bicarbonate ion which coordinates a base attack on the peptide bond followed by breakdown of a tetrahedral diol intermediate with the terminal amino acid as the leaving group [40]. Intriguingly, the S-LAP active site has diverged during Drosophila evolution replaced in many cases by amino acids incapable of Zn^2+ ^binding or bicarbonate coordination (Figure 4). In contrast, *granny-smith *contains the canonical active site residues. Furthermore, differences with likely functional relevance exist between the active site residues in S-LAP clusters I and II. The two members of Cluster II retain a high level of amino acid identity with the M17 aminopeptidase consensus, including i) 3 out of 4 conserved amino acids amongst the tight Zn^2+ ^binding residues (commonly referred to as "site 2"), ii) a conservative amino acid substitution (Lys327Gln) to a known M17 metal ligand amino acid at the fourth site and iii) a conservative amino acid substitution (Lys409Asp) to a known M17 metal ligand amino acid which contributes to divalent cation binding at the "loose" binding site (commonly referred to as "site 1"). This is in contrast to S-LAPs in Cluster I that possess i) limited or no similarity to the M17 aminopeptidase consensus and ii) an abundance of substitutions in residues that do not bind metal ions or are not characterized as M17 LAP metal ion ligands. Furthermore, 3 of the 6 S-LAPs in Cluster I have undergone a substitution at Lys339 which is essential for enzymatic activity [41]. The 7 characterized functional residues of the active site are displayed on the S-LAP protein alignment in Additional File 2, Figure S2.

### Purifying selection on the S-LAP gene family

Given the rapid expansion of the S-LAP gene family and the potential loss of catalytic activity amongst S-LAPs, we next sought to assess the role of positive selection in the functional diversification of the S-LAP gene family. To this end, we conducted codon based maximum likelihood analyses of selection for S-LAP Cluster I and II using all *Drosophila *S-LAP orthologs. This analysis resulted in strong evidence for purifying selection throughout the S-LAP gene. Analysis of all Cluster 1 sequences (including 68 gene sequences) resulted in a mean dN/dS estimate of 0.14, significant evidence of negative selection (p < 0.05) at 455 sites and no codons showing evidence of being under positive selection. Similarly, analysis of Cluster II (including 18 genes) resulted in a mean dN/dS estimate of 0.12, significant evidence of negative selection at 425 sites and no codons showing evidence of being under positive selection.

## Discussion

Advances in comparative genomics have revealed that the creation of novel genes is a widespread process [11,42-44] and that the composition of gene families associated with reproduction can vary dramatically between species [10]. Often, the expansion of gene families through gene creation results in functional diversification of gene family members, a process sometimes associated with rapid evolutionary changes at the DNA sequence and spatio-temporal expression levels. Furthermore, it has also been well documented that new retrogenes often acquire testis expression, however a limited amount is known about the specific functional ramifications of these phenomenon. To map specific functional consequences of gene family evolution, we have utilized comparative genomics to characterize the dynamic evolutionary history of the S-LAP gene family and documented S-LAP abundance and activity in spermatozoa.

### Gene family expansion and evolution

The recent availability of additional insect genome sequences has allowed the analysis of gene family evolution as it potentially relates to specific biological characteristics [45]. This approach has been of great utility in our studies of *Drosophila *sperm where gene creation has proven to be a widespread process and potentially a dynamic force in the molecular evolution of sperm [12]. Comparative genomic analyses of the sperm-specific S-LAPs in this study further extends this observation and demonstrates that the S-LAP gene families are highly heterogeneous in composition across the insect genomes that have been sequenced to date. This includes, but is not limited to, a dramatic expansion during the evolution of *Drosophila *(especially within S-LAP Cluster I) and variable copy numbers across three mosquito species. While some revision of the S-LAP gene family evolution may result from the availability of additional insect genomes, these results clearly reiterate the fundamental importance of gene creation during the evolution of reproductive traits in Drosophila.

### Leucyl aminopeptidases in *Drosophila *sperm

Aminopeptidases, a large family of related enzymes found throughout animal and plant kingdoms, have been described in many reproductive tissues including sperm and seminal fluids [46-48]. Our results extend these observations to include a newly recognized family, S-LAPs 1-8. Other than a role for a GPI-anchored aminopeptidase in the initiation of the acrosome activation in mussels (*Mytilus edulis*) [49], little functional data exists for leucylaminopeptidases either in sperm or indeed other aspects of reproductive biology. Our results provide strong circumstantial data suggesting that S-LAPs may play an important role in sperm function: (i) S-LAPs are expressed exclusively during spermatogenesis; (ii) they collectively represent the most abundant protein class in Drosophila sperm by a 2:1 mass ratio compared to tubulin (Figure 3; Table 2); (iii) bioinformatic analysis demonstrated S-LAPs as the most significantly over-represented protein domain within the proteome [33]; and (iv) analysis of stage specific expression during spermatogenesis (i.e. mitotic, meiotic and post-meiotic) revealed that all 8 S-LAPs were upregulated specifically during the meiotic and post-meiotic stages of late spermatogenesis (data not shown) [50], the developmental time window of spermiogenesis and sperm production. Further, we observed significant levels of leucyl aminopeptidase activity in both the soluble and insoluble fractions of purified sperm. S-LAP activity was recovered from the insoluble pellet in the presence of DTT. Furthermore, the two fractions differed in specific activities perhaps reflecting a partition of different S-LAPs that may be directly related to functional diversification between them (see below).

In contrast to the rapid evolution often observed in genes associated with male reproduction, we observe a strong signal of purifying selection across all S-LAPs suggesting a constrained functional role in sperm. This conservation is intriguing given that each cluster displays distinct patterns of substitutions at the active site consistent with functional diversification. Cluster I has undergone radical amino acid replacements in many of the canonical active site residues and all model studies of this class of enzyme suggest that these substitutions would be likely to render the catalytic site inactive. This observation, in conjunction with the evolutionary conservation (presumably due to functional constraint) of Cluster I, suggests the possible evolutionary acquisition of important functions independent of enzymatic activity. Comparison of Cluster I active site residues in *Drosophila *and mosquito reveals that most active site substitutions occurred prior to the evolutionary expansion of Cluster I, indicating that the Cluster I progenitor gene had potentially lost enzymatic activity prior to the expansion of this cluster (Figure 4). Future studies are needed to determine if the mosquito orthologs of Cluster I also function specifically in sperm.

In contrast, Cluster II S-LAPs more closely match the canonical M17 aminopeptidase active site and are likely to account for the enzymatic activity in purified sperm quantified in this study. The amino acid substitution (Arg413Val) in the catalytic site occurs at a residue not strictly required for enzymatic activity but instead is expected to decrease activity by 10 to 100-fold based on previous biochemical studies [51,52]. Inspection of the mosquito Cluster II active sites indicates that this substitution occurred more recently during *Drosophila *evolution. The other catalytic site, residue Lys339, is strictly conserved and has been shown essential for catalytic activity [41]. It is possible that the CG6372/CG32351 duplication event may have partially restored levels of enzymatic activity lost as a result of the Arg413Val substitution. Quantification of specific activity for each S-LAP is the next necessary step in assessing the functional diversification of the gene family.

## Conclusions

The presence of eight distinct S-LAPs and their extraordinarily high protein concentration in sperm strongly suggests that S-LAPs play a predominant (as yet unknown) role in *Drosophila *sperm biology. However, our results suggest that this role (or roles) cannot be linked to enzymatic activity in Cluster I S-LAPs where the canonical active site is clearly absent. An outstanding question therefore remains: what is the acquired functionality of Cluster I S-LAPs? One testable possibility is that Cluster I S-LAPs lost enzymatic activity but were retained, and subsequently diversified, following the acquisition of a structural function in sperm. While such a scenario for sperm protein evolution is unprecedented, it has been well documented in other enzyme families such as the crystallin family of lens proteins that evolved from ubiquitously expressed metabolic enzymes (reviewed in [53]). Although the precise evolutionary processes leading to the neofunctionalization of the crystallins are unknown, it did include the loss of enzymatic activity due to substitutions at critical active site residues, the expression of proteins at high concentrations and the subsequent establishment of their current structural and refractive roles in the eye lens [53]. It is noteworthy that the S-LAPs share extensive sequence homology (>40% at the amino acid level) to the bovine lens leucine aminopeptidase (data not shown). It will be of great interest to determine if, and to what extent, these two independent evolutionary processes parallel one another. Regardless, the S-LAP gene family represents a unique example wherein loss of function (and the presumed gain of a novel function) predates the dramatic expansion of a gene family. As such, neofunctionalization may itself lead to a situation where gene family expansion is selectively favorable, an observation that complements the general model where gene duplication predates and facilitates the evolution of novel functionality. Finally, in recent years characterized functions of LAPs have expanded to include a range of other cellular processes (e.g., transcriptional regulation, site-specific recombination and meiosis; reviewed in [54]). It will be of considerable interest to investigate whether S-LAPs have similarly acquired diversified roles in sperm function and physiology.

## Methods

### Comparative genomic analysis of the S-LAP family

BlastN was utilized to identify the closest paralog of the S-LAP gene family (Additional File 3), *granny-smith*, amongst annotated *D. melanogaster *genes and confirmed by estimates of synonymous divergence (dS). S-LAP orthology amongst *Drosophila *species was determined using reciprocal "best-hit" blastP against all annotated Drosophila proteins and was found to be consistent with current orthology annotation tables (http://flybase.org/). Identification of aminopeptidase genes related to the S-LAPs in other insects was conducted using a reciprocal blast approach where the eight S-LAPs were first compared to a combined database of RefSeq, non-RefSeq and ab initio coding sequences from *A. gambiae*, *C. quinquefasciatus*, *A. aegypti*, *T. castaneum*, *N. vitripennis*, *A. pisum *and *A. mellifera *using Tblastx. The second, reciprocal search compared all matches from the first search to all annotated *D. melanogaster *genes. Those that best matched members of the S-LAP family (in any order) were kept for further analysis. The same procedure was used to identify orthologs of S-LAP paralog, *granny-smith*. Concordant results, including a consistent estimate of S-LAP related gene copy number in each insect genome, were obtained using TblastN against the whole genome assembly for each species.

### Phylogenetic and evolutionary analysis

Alignments were conducted using CLUSTALW and phylogenetic analyses were conducted using the Minimum Evolution Method as implemented by MEGA4 [55]. The bootstrap consensus tree inferred from 1000 replicates is taken to represent the evolutionary history. Distances were calculated as the number of amino acid substitutions per site using the Poisson correction method. Consistent phylogenies were obtained using Bayesian phylogenetic methods [56]. Synonymous (dS) and nonsynonymous (dN) levels of divergence were estimated from "in-frame" CLUSTALW nucleotide alignments using the method of Nei and Gojobori [57]. Codon based analyses of selection were conducted using maximum-likelihood methods as implemented by HyPhy [58].

### RT-PCR analysis

Male carcasses (minus gonads) and testes were dissected, washed (3x) in phosphate buffered saline and flash frozen in liquid nitrogen. Total RNA was then isolated using manufacturer's protocols (Ambion195 RNAqueous Kit), and RNA was suspended in TE and quantified. Complementary DNA (cDNA) was carried out by standard protocols (Promega Improm-II Reverse Transcription System) using 75-ng input RNA (final concentration 3.75 ng/ul) from whole males (minus gonads) and testis. Gene-specific polymerase chain reaction (PCR) primers were used to amplify each S-LAP transcript from equal quantities of cDNA from the whole fly (minus gonads) and testis. Primer sequences are provided in Additional file 4. Equal volumes of each PCR reaction were visualized on 1% agarose gels.

### Sperm sample preparation and protein quantification

*D. melanogaster *sperm purified from Tempe-T strain males was solubilized over night at 25°C in CHAPS buffer containing 7 M Urea, 2 M Thiourea, 4% CHAPS, 33 mM DTT and 2.5 mM TCEP). The solubilized protein was separated from insoluble aggregates by ultracentrifugation, using a Beckmann-Coulter TL-100 and a TLA 100.2 fixed angle rotor for 30 min, 88,760 × g at 17°C. The supernatant was transferred in a new tube and stored at -80°C. Protein quantitation was measured using the RediPlate Protein Quantitation Kit (Molecular Probes) and calibration curves as per manufacturers instructions and fluorescence signals determined using a Fuji Laser Scanner FLA5000. Protein values were calculated using the Aida 2D-Densitometry software (Raytest, Straubenhardt, Germany).

### Protein precipitation for 2D gel electrophoresis

Sperm extracts were precipitated with 100% ice cold TCA (Sigma) at a final concentration of 20%. Following a 10 min incubation on ice, precipitated protein was collected by centrifugation for 5 min, 16 000 × g at 4°C. The supernatant was discarded and the pellet was washed three times with ice-cold acetone. After removing the acetone by a brief incubation at 37°C, the pellet was suspended in CHAPS buffer.

### 2D-Gel electrophoresis

The Multiphor II system from GE Healthcare was used for isoelectric focusing and SDS polyacrylamide gel electrophoresis following the manufacturers guidelines. Immobiline DryStrip gels 11 cm (pH 4-7, 6-11) and 24 cm (3-10NL) and precast 12.5% homogenous SDS gels were purchased from GE Healthcare. Immobiline Dry strip gels were rehydrated overnight in rehydration buffer (200 ml for 11 cm and 450 ml for 24 cm) containing 7 M Urea, 2 M Thiourea, 4% CHAPS, 18 mM DTT, 0.5% IPG buffer. pH4-7 and 3-10 NL Immobiline Dry strip gels were hydrated directly with the protein sample in rehydration buffer. Isoelectric focusing was performed at 20°C using the following 3-phase run parameters (upper limits for current and power were set to 2 mA and 2 W, respectively): phase 1, 300 V for 1 volt-hour (Vh); phase 2, a linear voltage increase to 3500 V over 2900 Vh; phase 3, constant 3500 V for 9.1 kVh (pH4-7) or 6.1 kVh (pH6-11). The isoelectric focusing protocol for the 24 cm 3-10NL; phase 1, 500 V for 1 Vh; phase 2, linear voltage increase to 3500 V over 3000 Vh; phase 3, constant 3500 V for 57 kVh. The strips were either frozen at -80°C or used immediately for second dimension gel electrophoresis. Before SDS gel electrophoresis strips were equilibrated for 15 min in 50 mM Tris pH 8.8, 6 M Urea, 30% Glycerol, 2% SDS, 0.002% bromphenolblue and 65 mM DTT to reduce disulfide bonds. A second equilibration step using the same reagents as above, but the DTT was replaced with 135 mM iodoacetamide. Iodoacteamide alkylates thiol groups prevents oxidation during electrophoresis. The Immobiline DryStrip gel was then transferred gel side down onto the SDS polyacrylamide gel. Gels were washed overnight in a 40% ethanol/10% acetic acid fixation solution. Proteins were visualized using Phastgel Blue Coomassie R350 (GE Healthcare) following manufacturer protocol.

### Mass spectrometry identification of S-LAPs

Eight S-LAPs were previously identified in the *Drosophila *sperm proteome by high throughput tandem mass spectrometry of whole sperm digests [18] and confirmed in a recent shotgun proteomic analysis [33]. The 2D-gel migration patterns of all eight S-LAPs were determined in an exhaustive study that identified the molecular identity of >80 major protein spots (U. Gerike and TLK, unpublished). Spots were excised from the gel and transferred into Microtiter plates, destained and digested using the Ettan Digester (GE Healthcare). The proteins were digested with sequencing grade trypsin (Promega) at 20 ng trypsin/ug sperm protein according to the manufacturers protocol. The resulting peptide mixtures were mixed with a saturated solution of a-cyano-4-hydroxycinnamic acid in 50% acetonitrile/0.1% trifluoroacetic acid, spotted and dried prior to analysis. Peptide masses were determined using a MALDI-TOF mass spectrometer (Micromass) running in linear mode. These results were further validated using tandem mass spectrometry (Orbitrap, Thermo Inc.). Peptides, prepared as described above, were subjected to tandem MS/MS and spectra analyzed using Scaffold (v2.02, Proteome Software, Portland, Oregon, USA). Peptide identifications were accepted if they could be established at greater than 95.0% probability as specified by the Peptide Prophet algorithm [59] and at least two tryptic peptides were detected. The average number of peptides identified per S-LAP protein was 5.8.

### Analyzing the MALDI TOF results

The Mowse scoring algorithm in a probabilistic framework of the Mascot Peptide Mass Fingerprint software (Matrix Science) was used as search tool. The search criteria were as follows: database - MSDB; organism - Drosophila; protease - Trypsin; fixed modification, Carbamidomethyl; variable modification - Oxidation (M); mass accuracy - 100 ppm and 0 missed cleavages.

### Relative protein quantification of gel spots

Three coomassie stained 2D-gels from *D. melanogaster *sperm in the pH 4-7 were scanned using FLA-5000 phosphoimager in digital mode and using a 472 nm excitation filter. Relative spot intensities were measured using version 3.23 of the AIDA 2D densitometry software (Raytest, Straubenhardt, Germany). The LAU (light absorbing units) were determined for each spot with background correction.

### Protein extraction and S-LAP activity assays

Sperm from 25 males of flies were incubated for 1 h at 25°C in 500 ml buffer A (50 mM HEPES pH7.4, 1 mM MnCl2, 67.5 mM DTT). Sperm was then broken up by sonication (~6s on ice) and the sample centrifuged 30 min at 16 000 g at 4°C. The supernatant was transferred to a new tube. The incubation mixture contained (in a total of 3 ml): 50 mM HEPES pH7.4, 1 mM MnCl2, 11.5 mM DTT and 100 mM L-Leu-AMC [Sigma]. S-LAP activity was determined by measuring the increase in fluorescence emission at 460 nm (340 nm excitation) using a Luminescence Spectrometer LS50B (Perkin Elmer). The reaction was started by the addition of the sperm extract. To calculate the units of activity a calibration curve was performed with 7-amino-4-methylcoumarin [Sigma] in the range 0-7 nmol.

## Competing interests

The authors declare that they have no competing interests.

## Authors' contributions

TLK and SD conceived the experiments and wrote the paper and ECW performed the PCR experiments. All authors read and approved the final manuscript.

## Supplementary Material

Additional file 1 **Complete S-LAP phylogeny**. This file includes a complete S-LAP phylogeny with all *Drosophila *orthologs. Click here for file

Additional file 2 **S-LAP protein alignment**. This file includes the S-LAP protein alignment denoting the seven characterized functional residues of the active site. Click here for file

Additional file 3 **S-LAP primers**. This file includes a table listing the primers used in S-LAP RT-PCR experiments. Click here for file

Additional file 4 **Name and symbol key**. This file includes a table listing the names and symbols of the genes encoding aminopeptidases referred to in this study. Click here for file
