# Supplementary figures and images for: Expansion and functional diversification of a leucyl aminopeptidase family that encodes the major protein constituents of Drosophila sperm
Source: BMC Genomics. 2011 Apr 5;12:177. doi: 10.1186/1471-2164-12-177 (PMC3078892; doi:10.1186/1471-2164-12-177)

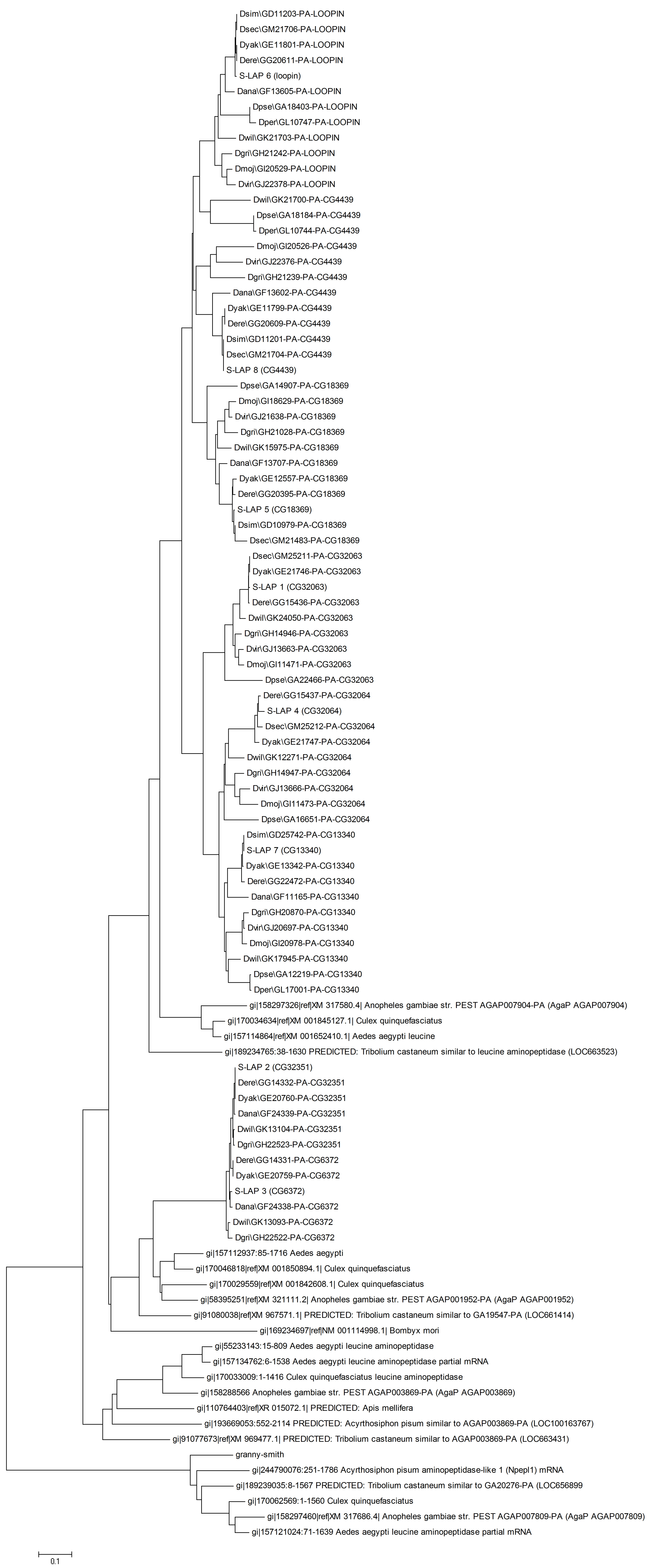

Supplement: Additional file 1 — Complete S-LAP phylogeny. This file includes a complete S-LAP phylogeny with all Drosophila orthologs. [file 1471-2164-12-177-S1.JPEG]

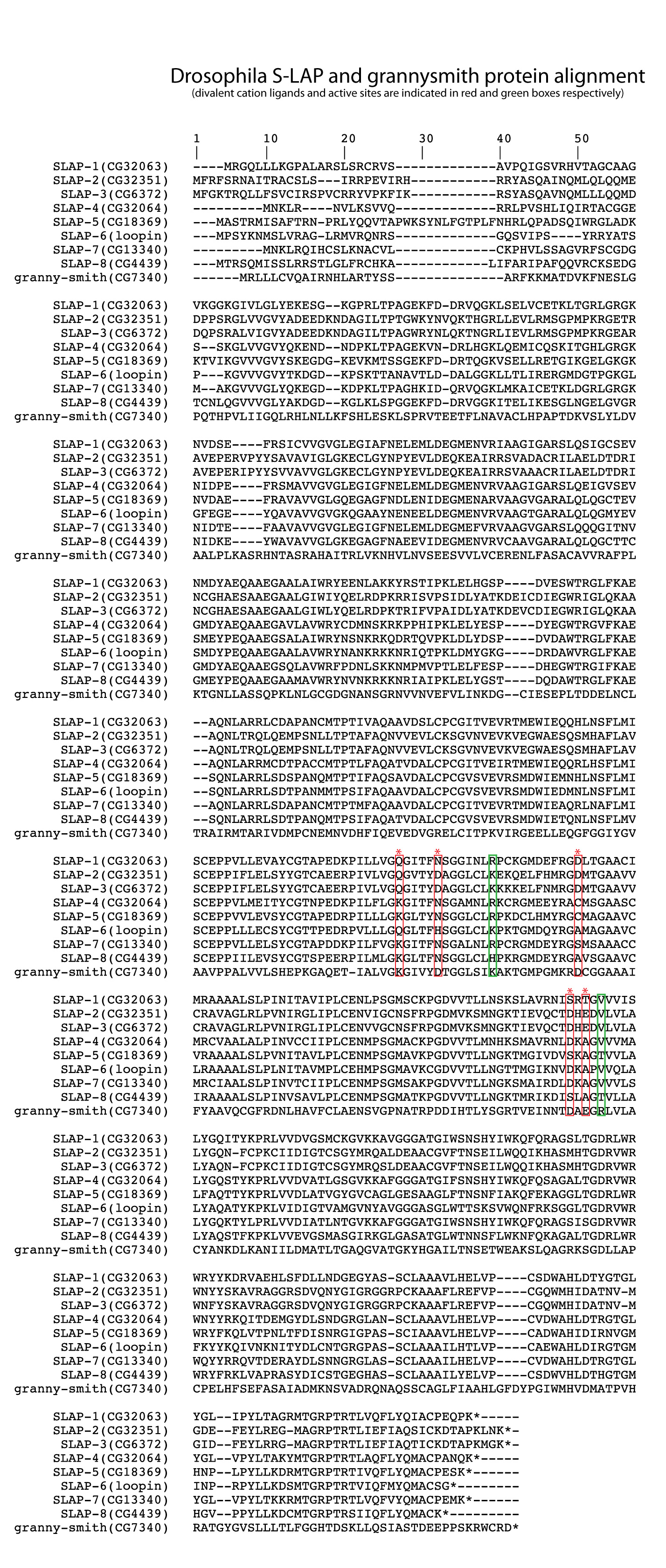

Supplement: Additional file 2 — S-LAP protein alignment. This file includes the S-LAP protein alignment denoting the seven characterized functional residues of the active site. [file 1471-2164-12-177-S2.JPEG]
