# Supplementary material for: Expansion and functional diversification of a leucyl aminopeptidase family that encodes the major protein constituents of Drosophila sperm
Source: BMC Genomics. 2011 Apr 5;12:177. doi: 10.1186/1471-2164-12-177 (PMC3078892; doi:10.1186/1471-2164-12-177)
Supplement: Additional file 3 — S-LAP primers. This file includes a table listing the primers used in S-LAP RT-PCR experiments. [file 1471-2164-12-177-S3.DOCX]

**Additional File 3. Names and Symbols**

| **Genes/Protein** | | |
| --- | --- | --- |
| **Flybase ID** | **Gene Symbol** | **Gene Name** |
| FBgn0035915 | *CG6372* | *Sperm Leucyl Aminopeptidase 1* |
| FBgn0035915 | *CG32351* | *Sperm Leucyl Aminopeptidase 2* |
| FBgn0045770 | *CG32063* | *Sperm Leucyl Aminopeptidase 3* |
| FBgn0052064 | *CG32064* | *Sperm Leucyl Aminopeptidase 4* |
| FBgn0033860 | *CG18369* | *Sperm Leucyl Aminopeptidase 5* |
| FBgn0259795 | *CG4750* | *Sperm Leucyl Aminopeptidase 6; (loopin-1)* |
| FBgn0033868 | *CG13340* | *Sperm Leucyl Aminopeptidase 7* |
| FBgn0034132 | *CG4439* | *Sperm Leucyl Aminopeptidase 8* |
| FBgn0040493 | CG7340 | granny-smith |
