# Supplementary material for: Expansion and functional diversification of a leucyl aminopeptidase family that encodes the major protein constituents of Drosophila sperm
Source: BMC Genomics. 2011 Apr 5;12:177. doi: 10.1186/1471-2164-12-177 (PMC3078892; doi:10.1186/1471-2164-12-177)
Supplement: Additional file 4 — Name and symbol key. This file includes a table listing the names and symbols of the genes encoding aminopeptidases referred to in this study. [file 1471-2164-12-177-S4.DOCX]

**Additional File 4. RT-PCR Primers**

| **Gene** | **Forward (5' to 3')** | **Reverse (5' to 3')** |
| --- | --- | --- |
| *CG18369*  *S-LAP 5* | TCACCTTCGACATCAGCAAT | ATCTGATACAGGAACTGTAC |
| *CG4439*  *S-LAP 8* | TGTGGACCAATAACTCTTTC | ATGCAATCCTTGAGCAAATA |
| *CG6372*  *S-LAP 1* | GTTGTGGAACTTCTACAGCAA | AGATCGTCTGTGCAATGAATT |
| *CG32063*  *S-LAP 3* | ACTGGTATTTGGTCGAATTCTCA | ATCAATCCATATGTGCTCAATAGT |
| *CG32064*  *S-LAP 4* | CATTGTGGAATTATTATAGGAA | ATGTACTTTGCGGTGAGATA |
| *CG32351*  *S-LAP 2* | CTGTGGAACTACTACAGCAA | ATAAACTCGATGAGTGTCCTT |
| *CG4750*  *S-LAP 6* | TGTTCAAGTACTACAAACAGA | TACATGAACTGGATAACAGT |
| *CG13340*  *S-LAP 7* | CAACTCACACTACATCTGGAAG | TCATACGCTTCTTAGTTAGATA |
